# Diarylheptanoid Phytoestrogens Isolated from the Medicinal Plant *Curcuma comosa*: Biologic Actions *in Vitro* and *in Vivo* Indicate Estrogen Receptor–Dependent Mechanisms

**DOI:** 10.1289/ehp.0900613

**Published:** 2009-03-23

**Authors:** Wipawee Winuthayanon, Pawinee Piyachaturawat, Apichart Suksamrarn, Mathurose Ponglikitmongkol, Yukitomo Arao, Sylvia C. Hewitt, Kenneth S. Korach

**Affiliations:** 1 Department of Physiology, Faculty of Science, Mahidol University, Bangkok, Thailand; 2 Laboratory of Reproductive and Developmental Toxicology, National Institute of Environmental Health Sciences, National Institutes of Health, Department of Health and Human Services, Research Triangle Park, North Carolina, USA; 3 Department of Chemistry, Faculty of Science, Ramkhamhaeng University, Bangkok, Thailand; 4 Department of Biochemistry, Faculty of Science, Mahidol University, Bangkok, Thailand

**Keywords:** Curcuma comosa, diarylheptanoid, ER dependent, ERE dependent, ERE independent, estrogen-regulated genes, uterotrophic activity

## Abstract

**Background:**

Diarylheptanoids isolated from *Curcuma comosa* Roxb. have been recently identified as phyto estrogens. However, the mechanism underlying their actions has not yet been identified.

**Objectives:**

We characterized the estrogenic activity of three active naturally occurring diarylheptanoids both *in vitro* and *in vivo*.

**Methods:**

We characterized mechanisms of estrogenic action of the diarylheptanoids (3*S*)-1,7-diphenyl-(6*E*)-6-hepten-3-ol (D1), 1,7-diphenyl-(6*E*)-6-hepten-3-one (D2), and (3*R*)-1,7-diphenyl-(4*E*,6*E*)-4,6-heptadien-3-ol (D3) by using a real-time polymerase chain reaction assay, a mammalian transfection model, and a uterotrophic assay in mice.

**Results:**

All diarylheptanoids up-regulated estrogen-responsive genes in estrogen-responsive breast cancer cells (MCF-7). In HepG2 cells transfected with estrogen receptor (ER) β or different ERα functional receptor mutants and the Vit-ERE-TATA-Luc reporter gene, all diarylheptanoids induced transcription through a ligand-dependent human ERα-ERE–driven pathway, which was abolished with ICI 182,780 (ER antagonist), whereas only D2 was active with ERβ. An ERα mutant lacking the functional AF2 (activation function 2) region was not responsive to 17β-estradiol (E_2_) or to any of the diarylheptanoids, whereas ERα lacking the AF1 domain exhibited wild-type–like activity. D3 markedly increased uterine weight and proliferation of the uterine epithelium in ovariectomized mice, whereas D1 and D2 were inactive. D3, like E_2_, up-regulated lactoferrin (*Ltf*) gene expression. The responses to D3 in the uterus were inhibited by ICI 182,780. In addition, D3 stimulated both classical (*Aqp5*) and nonclassical (*Cdkn1a*) ER-mediated gene regulation.

**Conclusions:**

The results suggest that the D3 diarylheptanoid is an agonist for ER both *in vitro* and *in vivo*, and its biological action is ERα selective, specifically requiring AF2 function, and involves direct binding via ER as well as ERE-independent gene regulation.

Medicinal herbal products have traditionally been used as home remedies for treatment of many diseases for centuries (Rodriguez-Fragoso et al. 2008). Although many have promising therapeutic potential, in general there is a lack of rigorous controlled scientific testing, and little information is available regarding appropriate patient use or adverse effects. Biosafety and efficacy are major public concerns, particularly in developing countries, which are still establishing biosafety policies. However, the extensive traditional uses of nonapproved herbal medicines, as well as environmental exposures, result in a possible risk of both acute and chronic toxicities. Chronic forms of toxicities such as carcinogenicity, mutagenicity, and hepatotoxicity have been reported (Pak et al. 2004). In addition, some specific patient groups may have more of a health risk (e.g., pregnant or nursing mothers and the elderly). Herbal medicines may also interfere with the efficacy of conventional medicines through herb–drug interactions (Tomlinson et al. 2000). Many herbs that have traditionally been reported to have estrogen-like activity are potentially abortifacient (Sylvestre et al. 2006), which may affect certain groups of individuals.

Phytoestrogens, plant-derived nonsteroidal polyphenolic compounds, are present in soybeans, clover, and oilseeds. Isoflavones, lignans, coumestans, resorcylic acid lactones, and stilbenes are known phyto estrogens (Knight and Eden 1996). Isoflavones, such as genistein, apigenin, and kaempferol, have stronger binding affinity for estrogen receptor (ER) β than for ERα (Kuiper et al. 1998). However, the transcriptional activity of phytoestrogens such as genistein, daidzein, apigenin, naringenin, kaempferol, and coumestrol can be mediated by either ERα or ERβ (Harris et al. 2005). In addition, the stilbene resveratrol, a phytoestrogen from grapes, induced the estrogen-regulated gene progesterone receptor (*PR*) in MCF-7 (human breast cancer) cells in a dose-dependent manner (Gehm et al. 1997). These estrogen-like activities of phytoestrogens have been demonstrated to have beneficial roles in prevention of several diseases, including cardiovascular diseases, osteoporosis, cancers, and menopausal symptoms (Usui 2006). However, phytoestrogen had no significant effect on hot flashes in women (Huntley and Ernst 2004). Therefore, the search for novel phytoestrogens as an alternative treatment is still a subject of high interest for postmenopausal women.

Diarylheptanoids consist of two aromatic rings linked by a linear seven-carbon aliphatic chain. The first naturally occurring diarylheptanoid, curcumin, a yellow dye of the *Curcuma* species, was reported in the early 1800s (Keserü and Nógrádi 1995). Diarylheptanoids have been reported to have a variety of therapeutic actions *in vitro* such as inhibition of platelet aggregation (Doug et al. 1998), antioxidation (Yao et al. 2007), antiinflammation (Sodsai et al. 2007), and antitumor-promoting effects (Chun et al. 1999; Lee et al. 1998). Because *Curcuma comosa* Roxb., an indigenous medicine, has estrogen-like activity (Piyachaturawat et al. 1995a, 1995b) and has been extensively used among menopausal women, it is essential to characterize its biological action to avoid any risk that may occur from chronic consumption or environmental exposures. Recently, diarylheptanoids isolated from *C. comosa* have also been reported to exhibit estrogenic activity both *in vitro* (Suksamrarn et al. 2008) and *in vivo* (Winuthayanon et al. 2009) and are useful for nutraceutical health promotion and hormone replacement therapy. The reported high content of diarylheptanoids in *C. comosa* (Suksamrarn et al. 2008) provided this plant species as an attractive source of novel phytoestrogens. However, the precise mechanisms of the estrogenic action of diarylheptanoids have not yet been identified. The diarylheptanoids (3*S*)-1,7-diphenyl-(6*E*)-6-hepten-3-ol (D1), 1,7-diphenyl-(6*E*)-6-hepten-3-one (D2), and (3*R*)-1,7-diphenyl-(4*E*,6*E*)-4,6-heptadien-3-ol (D3; Figure 1), the major components of *C. comosa*, exhibited high estrogenic activity (Suksamrarn et al. 2008). In the present study we aimed to characterize the estrogenic activity of these potent naturally occurring diarylheptanoids *in vivo* and determine their mechanism of action on ERs *in vitro* by using well-characterized and appropriate experimental model systems.

## Materials and Methods

### Chemicals and compounds

We obtained DNA restriction and modification enzymes from New England Biolabs Inc. (Beverly, MA, USA) and polymerase chain reaction (PCR) reagents from Promega (Madison, WI, USA). We purchased 17β-estradiol (E_2_), DMSO, flutamide, and sesame oil from Sigma (St. Louis, MO, USA). All chemicals were dissolved in ethanol (EtOH) unless otherwise indicated. ICI 182,780 (ICI) was purchased from Tocris Bioscience (Ellisville, MS, USA) and dissolved in DMSO. The diarylheptanoids used in this study (D1, D2, and D3) were isolated from *C. comosa* as described previously by Suksamrarn et al. (2008). We checked the purity of these compounds by thin-layer chromatography and nuclear magnetic resonance spectroscopy.

### Plasmids

The mammalian expression plasmids for human ERα (hERα) and hERβ (pcDNA3-hERα and pcDNA3-hERβ) have been described previously (Hall and Korach 2002). The expression plasmids for mouse ERα wild-type (pcDNA3-mERαWT) and AF1 domain deleted mouse ERα (mERKOE1; pcDNA3-mERKOE1) have been previously described (Couse et al. 1995). To make the pcDNA3-mAF2ER plasmid, the leucine residues corresponding to amino acids 543 and 544 of mouse ERα were replaced with alanine by using QuikChange Site-Directed Mutagenesis Kit (Stratagene, La Jolla, CA, USA) according to the manufacturer’s instructions. We used the pcDNA3-mERαWT plasmid as a template and reacted it with the oligonucleotide primers: 5′-GTG CCC CTC TAT GAC GCG GCT CTA GAG ATG TTG GAT GCC CAC-3′ and 5′-GTG GGC ATC CAA CAT CTC TAG CTC CGC GTC ATA GAG GGG CAC-3′. The mutated clone was confirmed by sequencing. The 3×-Vit-ERE-TATA-Luc plasmid was a gift from D.P. McDonnell (Duke University Medical Center, Durham, NC, USA).

### Uterine bioassay in adult wild-type (WT) ovariectomized mice

Animals were handled according to National Institute of Environmental Health Sciences (NIEHS) Animal Care and Use Committee guidelines and in compliance with an NIEHS-approved animal protocol; all animals were treated humanely and with regard for alleviation of suffering. Adult female C57BL6J mice, approximately 10 weeks of age, were either purchased from Charles River Laboratories (Raleigh, NC, USA) or generated at the NIEHS. All mice were ovariectomized (OVX) and housed for 10–14 days to eliminate endogenous ovarian steroids before the study. Animals were treated with sesame oil (vehicle), 2.5 mg diarylheptanoids (D1, D2, or D3), or 0.25 μg E_2_ in 100 μL sesame oil (body weight, ~ 25 g per mouse) by subcutaneous administration. Some animals were treated with 45 μg ICI or 2 mg flutamide [androgen receptor (AR) antagonist] dissolved in 50 μL DMSO injected intraperitoneally 30 min before E_2_ or diarylheptanoid injection. Animals were treated every 24 hr for 3 consecutive days. Animals were sacrificed 24 hr after the last injection by using CO_2_ asphyxiation, and the uteri were collected, blotted, and weighed. A portion of each uterus was fixed in 10% formalin, embedded in paraffin, and cross-sectioned. We detected Ki67 as previously described (Hewitt et al. 2006). The remaining uterine samples were snap frozen in liquid nitrogen for later RNA isolation using Trizol reagent (Invitrogen, Carlsbad, CA, USA) according to the manufacturer’s protocol.

### Nonclassical ERE-independent gene analysis in knock-in/knockout (KI/KO) mice

Adult female C57BL6J ERα knockout (αERKO) mice (Lubahn et al. 1993) were generated at Taconic Farms (Germantown, NY, USA). The KI/KO (heterozygous male nonclassical ER knock-in NERKI^(–/+)^ × female αER^(–/+)^) mice were generated at Charles River (Wilmington, MA, USA) as described previously (Jakacka et al. 2002). All mice used were ovariectomized and housed for 10–14 days. Wild-type (WT), αERKO, and KI/KO OVX mice were treated with sesame oil (vehicle), 2.5 mg D3 (the most potent among the three diarylheptanoids on uterotrophic action), or 0.25 μg E_2_ in 100 μL sesame oil, by subcutaneous administration. Animals were sacrificed 2 hr after treatment, and uteri were collected and snap frozen for later RNA isolation.

### Cell culture conditions

MCF-7 human breast cancer cells, which express ERs, were grown in phenol red–free Dulbecco’s modified Eagle medium/Nutrient Mix F12 (DMEM/F12) media containing 10% fetal bovine serum (FBS; Atlanta Biologicals, Lawrenceville, GA, USA), 100 U/mL penicillin, and 100 μg/mL streptomycin. HepG2 (human hepatocellular carcinoma) cells, which do not express ERs, were grown in phenol red–free minimum essential medium (MEM) containing 2 mM l-glutamine, 10% FBS, 100 U/mL penicillin, and 100 μg/mL streptomycin. We purchased culture reagents from Invitrogen unless otherwise indicated.

### MCF-7 cell treatment and RNA isolation

MCF-7 cells were plated in six-well plates at a density of 1 × 10^6^ cells/well overnight in DMEM/F12 containing 10% FBS. Cells were then washed twice with phosphate-buffered saline (PBS), and 2 mL DMEM/F12 containing 10% dextran coated-charcoal FBS [stripped FBS (SFBS; Hyclone, South Logan, UT, USA] was added into each well. After 24 hr, cells were treated with EtOH (vehicle), 1 nM E_2_, or various concentrations of diarylheptanoids in the presence or absence of 1 μM ICI for 24 hr. We isolated the total RNA from MCF-7 cells using the RNeasy Mini Kit (Qiagen, Valencia, CA, USA) according to the manufacturer’s protocol. We then reverse transcribed 2 μg total RNA using the SuperScript First-Strand Synthesis System (Invitrogen).

### Real-Time PCR

We synthesized cDNA from MCF-7 cells and mouse uteri and obtained cycle time (Ct) values by real-time PCR with SYBR Green I dye using the ABI PRISM 7900 Sequence Detection System and analysis software (Applied Biosystems, Foster City, CA, USA) as previously described (Hewitt et al. 2005). Primer sequences were created using Applied Biosystems Primer Express Software (version 2.0) or were purchased from Sigma-Genosis (St. Louis, MO, USA); the sequences for primers are shown in Table 1. We determined the expression ratios relative to vehicle control and normalized them to a ribosomal protein L7 gene (*Rpl7*) for uterine samples and beta-2-micro globulin gene (*B2M*) for samples from MCF-7 cells by quantification of cDNA according to the mathematical model described by Pfaffl (2001).

### Transient transfection and luciferase assay in HepG2 cells

HepG2 cells were plated in 24-well plates at a density of 2 × 10^5^ cells/well in MEM containing 10% SFBS and incubated overnight. Cells were washed twice with PBS, and then 500 μL MEM with 10% SFBS was added to each well. Cells were transfected for 6 hr with 0.1 μg/μL pcDNA3-hERα, pcDNA3- hERβ, pcDNA3-mERαWT, pcD-NA3-mERKOE1, pcDNA3-mAF2ER, 3×-Vit-ERE-TATA-Luc, or pRL-TK (constitutively active *Renilla* reporter plasmid; Promega) by using FuGENE6 transfection reagent (Roche Applied Science, Indianapolis, IN, USA). Afterward, we treated cells for 20 hr with 1 nM E_2_ or various concentrations of diarylheptanoids in the presence or absence of 1 μM ICI. We then assayed HepG2 cells for luciferase activity using the Dual-Luciferase reporter assay system (Promega) and an LMAXII luminometer (Molecular Devices, Sunnyvale, CA, USA).

### Statistical analysis

We assessed data sets for statistical significance (*p* < 0.05) using Jmp Software, version 5.0 (SAS Institute Inc., Cary, NC, USA). All data were assessed for statistically significant differences via a one-way analysis of variance followed by the Tukey-Kramer post hoc test.

## Results

### ER-Dependent Estrogenic Activity *in Vitro*

#### Diarylheptanoids stimulate estrogen-regulated genes in MCF-7 cells

**Figure 2:**
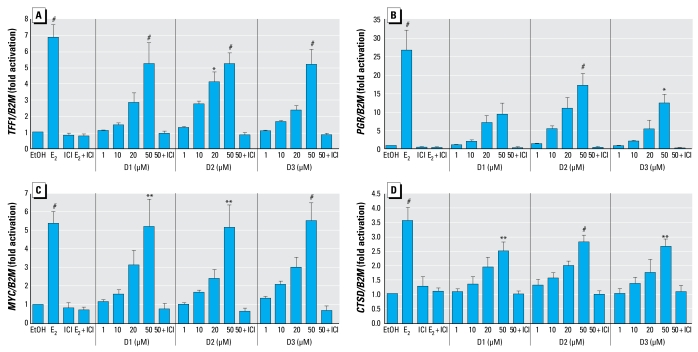
Endogenous gene expression in MCF-7 cells treated with EtOH (vehicle), 1 nM E_2_, or 1, 10, 20, or 50 μM diarylheptanoid (D1, D2, or D3) in the presence or absence of 1 μM ICI for 24 hr. Fold activation of *TFF1* (*A*), *PGR* (*B*), *MYC* (*C*), and *CTSD* (*D*) compared with EtOH. Each value represents the average increase above EtOH obtained from three independent experiments performed in duplicate. ^*^p < 0.05, ^**^p < 0.01, and ^#^*p* < 0.001, compared with EtOH.

To elucidate the specificity of the estrogenic response, we assessed the activity of diarylheptanoid on the expression of estrogen-regulated genes: *TFF1* (or *pS2*) (May and Westley 1997), progesterone receptor (*PGR*) (Soto et al. 1995), *MYC* (Dubik et al. 1987), and cathepsin D (*CTSD*) (Westley and May 1987), which are known estrogen-inducible genes in MCF-7 cells. As shown in Figure 2, we determined a dose–response effect in which 50 μM of D1, D2, and D3 significantly increased the mRNA expression of *TFF1*, *MYC*, and *CTSD* to a level comparable to that induced by 1 nM E_2_. However, at a dose of 50 μM, D2 and D3 significantly increased *PGR* expression, but D1 was weak and its effect was not statistically different from that of EtOH. The responses of all diarylheptanoids (D1, D2, and D3) and E_2_ were fully inhibited in the presence of 1 μM ICI.

#### Diarylheptanoids require the AF2 (activation function 2) region of ER for transactivation function

**Figure 3:**
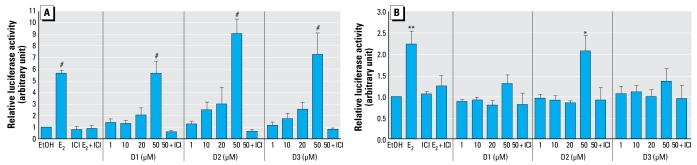
Relative luciferase activity in HepG2 cells transfected with hERα (*A*), hERβ (*B*), and 3 ×-Vit-ERE-TATA-Luc plasmids that we treated with EtOH (vehicle), 1 nM E_2_, or 1, 10, 20, 50 μM diarylheptanoid (D1, D2, or D3) in the presence or absence of 1 μM ICI. Each value was obtained from three independent experiments performed in triplicate. ^*^p < 0.05, ^**^p < 0.01, and ^#^*p* < 0.001, compared with EtOH.

We examined the molecular mechanism of ER-dependent gene regulation by diaryl heptanoid. HepG2 cells that do not express ERs were transiently transfected with plasmids containing either hERα or hERβ and an estrogen-responsive element (ERE)-fused luciferase reporter plasmid (3×-Vit-ERE-TATA-Luc) (Figure 3). In the presence of hERα, diarylheptanoids (D1, D2, and D3) stimulated ERE-mediated luciferase activity in a dose-dependent manner, with the maximum relative luciferase activity at a dose of 50 μM comparable to the activity of 1 nM E_2_ (Figure 3A). Coincubation with 1 μM ICI inhibited the transactivation of 3×-Vit-ERE-TATA-luc plasmid by diarylheptanoids and E_2_. In the presence of hERβ, only 50 μM D2 or 1 nM E_2_ significantly stimulated the luciferase activity (Figure 3B). However, neither D1 nor D3 at any concentration stimulated the ERβ-mediated luciferase trans-activation, suggesting that D1 and D3 can activate their estrogenic actions through ERα but not through ERβ, whereas D2 can exert its transcriptional activity through either ERα or ERβ.

**Figure 4:**
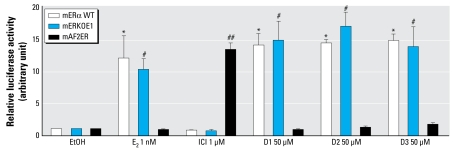
Relative luciferase activity in HepG2 cells transfected with mERαWT (WT mouse ERα), mERKOE1 (deleted AF1 region of mouse ERα), mAF2ER (mutated AF2 region of mouse ERα), and 3×-Vit-ERE-TATA-Luc plasmids that were treated with EtOH (vehicle), 1 nM E_2_, 1 μM ICI, or 50 μM D1, D2, or D3. Each value was obtained from three independent experiments performed in triplicate. ^*^p < 0.05, compared with EtOH for mERαWT group. ^#^*p* < 0.05, compared with EtOH for mERKOE1 group. ^##^*p* < 0.05, compared with EtOH for mAF2ER group.

To evaluate the effect of diarylheptanoids on specific functional domains of ER, we cotransfected plasmids expressing mutated ER with ERE-dependent luciferase reporters (3×-Vit-ERE-TATA-Luc) in HepG2 cells. In the presence of AF1 domain-deleted mouse ERα (mERKOE1) plasmid, 50 μM diarylheptanoids (D1, D2, and D3) and 1 nM E_2_ significantly stimulated ERE-dependent luciferase activity in a manner similar to that of WT ERα (mERαWT) (Figure 4). Unlike the AF1 mutation, diarylheptanoids (D1, D2, and D3) and E_2_ both failed to stimulate ERE-dependent luciferase activity of the mouse ERα containing two-point mutations in its AF2 domain (mAF2ER). However, 1 μM ICI significantly stimulated the ERE-mediated luciferase activity of the mAF2ERα in a manner described previously by Mahfoudi et al. (1995).

### ER-Dependent Estrogenic Activity *in Vivo*

#### Estrogenic activity of diarylheptanoids was also evaluated in vivo using a 3-day uterine bioassay in mice

**Figure 5:**
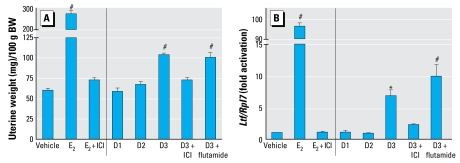
Uterine wet weight (mg/100 g body weight (BW); *A*] and uterine *Ltf* expression (*B*) in adult OVX mice treated for 3 consecutive days with sesame oil (vehicle), 0.25 μg E_2_, or 2.5 mg diarylheptanoids (D1, D2, or D3). In addition, 45 μg ICI (ER antagonist) or 2 mg flutamide (AR antagonist) were coadministered with 2.5 mg D3. ^*^p < 0.05, and ^#^*p* < 0.001, compared with vehicle ( *n* = 3–4 animals/group).

Uterine weights were significantly increased, approximately 1.8-fold, by 2.5 mg D3 compared with a 4.6-fold increase seen with 0.25 μg E_2_, the positive control (Figure 5A). However, D1 and D2 had no effect on uterine weight. Treatment with 45 μg ICI essentially abolished the increase in uterine weight in both D3- and E_2_-treated groups. To test whether the uterotrophic activity of D3 may be mediated through the AR (Hewitt et al. 2006), we cotreated mice with 2 mg flutamide (AR antagonist) and D3. The results showed that flutamide did not significantly alter the action of D3 in mouse uterus. Expression of the uterine estrogen-inducible gene lactoferrin (*Ltf*) was increased by D3 and E_2_ (6.8- and 96.6-fold, respectively). ICI inhibited the D3- or E_2_-mediated induction of *Ltf* (Figure 5B). Flutamide did not alter the D3-mediated change in *Ltf* expression; the level of expression was approximately 9.9-fold.

**Figure 6:**
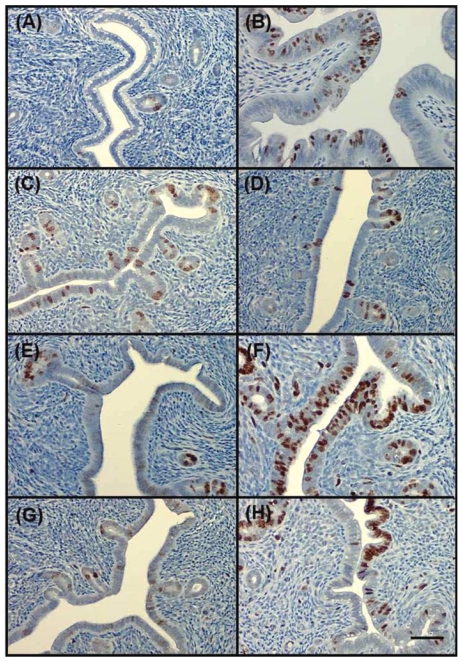
Ki67 immunohistochemistry. Cross-sectional uterine tissues from adult OVX mice treated 3 consecutive days with sesame oil (vehicle; *A*), 0.25 μg E_2_( *B*), 45 μg ICI + E_2_( *C*), 2.5 mg D1( *D*), 2.5 mg D2 (*E*), 2.5 mg D3 (*F*), 45 μg ICI + 2.5 mg D3 (*G*), and 2 mg flutamide + 2.5 mg D3 (*H*). Tissues are stained with an antibody to the proliferative marker Ki67. Bar = 40 μm.

To evaluate the effect of the compounds on uterine epithelial proliferation, we analyzed Ki67, a marker for proliferation (Gerdes et al. 1984), by immunohistochemistry. Ki67 was increased in uterine epithelia of mice treated with 2.5 mg D3 and 0.25 μg E_2_, as well as those cotreated with 2.5 mg D3 and 2 mg flutamide, compared with vehicle control (Figure 6). We detected no staining in those mice that received D1, D2, and ICI.

#### Classical and ERE-independent gene activation in vivo

**Figure 7:**
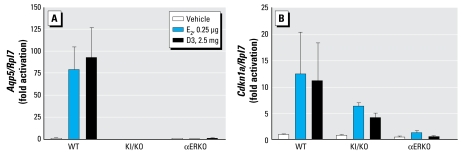
Effect of D3 on the classical (ERE-mediated) and nonclassical genes in mouse uteri. *Aqp5* (*A*), and *Cdkn1a* (*B*) in mouse uteri from adult WT, αERKO, and KI/KO OVX mice treated for 2 hr with vehicle (sesame oil), 0.25 μg E_2_, or 2.5 mg D3. Data represent the mean ± SE increase above the vehicle group, with expression ratios normalized to *Rpl7* by real-time reverse transcriptase PCR (*n* = 3–7 animals/group).

To elucidate whether the uterotrophic activity of D3, the most potent estrogenic compound among the diarylheptanoid analogues in the uterus, can use the nonclassical ERE-independent pathway, we compared gene response in KI/KO, αERKO, and WT OVX mice because KI/ KO mice selectively use the ERE-independent mechanism of ER (O’Brien et al. 2006). We determined the expression of an ERE-dependent gene (aquaporin 5; *Aqp5*) and an ERE-independent gene (*Cdkn1a* or p21) (O’Brien et al. 2006). Treatment for 2 hr with 2.5 mg D3 or 0.25 μg E_2_ did not alter the expression of *Aqp5* in either KI/KO or αERKO mice (Figure 7A). However, D3 and E_2_ markedly increased the expression of *Aqp5* in WT mice compared with vehicle control. In addition, the results showed that *Cdkn1a* was up-regulated similarly in both WT and KI/KO mice by D3 or E2 treatment (Figure 7B). These findings suggest that diarylheptanoid (D3) stimulates gene expression in the uterus through both classical and tethered ERα-mediated pathways.

## Discussion

In this study we characterized the estrogenic activity of diarylheptanoids, naturally occurring compounds from *C. comosa*, in established *in vitro* and *in vivo* model systems. Diarylheptanoids (D1, D2, and D3) stimulated several endogenous estrogen-regulated genes in MCF-7 cells in an ER-dependent manner. Transcriptional activation of a reporter gene required a functional AF2 region of the ER protein. Moreover, we demonstrated that only the D3 compound exerted ER-dependent uterotrophic activity in WT OVX mice. Additionally, the estrogenic activity of D3 in the uterus was mediated through both classical and nonclassical ERE-independent mechanisms. To our knowledge, this is the first study showing the molecular mechanism and selectivity of these diarylheptanoids as novel phytoestrogens.

We explored the specificity of diarylheptanoids on several known estrogen-regulated endogenous genes in MCF-7 cells, a well- established estrogen-sensitive cell model used to assay the estrogenicity of putative estrogenic chemicals (Jorgensen et al. 2000). E_2_ has been shown to up-regulate *TFF1* (May and Westley 1997), *PGR* (Nardulli et al. 1988), *MYC* (Dubik et al. 1987), and *CTSD* (Westley and May 1987) in breast tissues. These genes contain nonconsensus ERE sequences on their promoters (Berry et al. 1989; Dubik and Shiu 1992; Petz and Nardulli 2000; Wang et al. 1997). In the present study, D1, D2, and D3 induced the expression of almost all these genes, whereas D1 weakly increased the expression of *PGR*. The effects were ER dependent because they were inhibited by ICI. Therefore, diarylheptanoids induced these E_2_-regulated endogenous genes in MCF-7 cells in a manner similar to other reported estrogen agonists.

Both ERα and ERβ are able to bind to a number of structurally diverse compounds (Kuiper et al. 1998); here, using an ERE-driven luciferase assay to assess diarylheptanoid-induced transactivation, we demonstrated that the diarylheptanoids D1 and D3 induced ERE-dependent luciferase activity only in the presence of hERα, and not hERβ. Based on previous reports of ERβ selectivity for xeno estrogen binding (Kuiper et al. 1998), it was surprising that D1 and D3 stimulated no activity with ERβ. The difference may be that our study is a direct functional assessment of receptor-mediated actions versus a ligand-binding activity, which does not necessary reflect biological activity. Our study indicated that D1 and D3 had selectivity toward hERα/ERE-driven transcription. However, D2 had no selectivity for a specific ER form because D2 mediated transcription through either ERα or ERβ. This may reflect subtle differences in the ligand binding pocket of ERα and ERβ for D1 and D3.

Transcriptional activity of estrogenic compounds depends on the cellular and promoter contexts that are mediated by two different activation functions of the ER: AF1 and AF2 (Tzukerman et al. 1994). In most cases, AF1 works synergistically with AF2 to activate transcription through coregulatory factors such as TIF2 (p160 coactivator family) (Benecke et al. 2000). We found that the AF1 region of ER was dispensable for transcriptional activity of all three diarylheptanoids (D1, D2, and D3) as well as E_2_ in the HepG2 cell model. Several phosphorylation sites have been identified in the A/B domain/AF1 region of the N-terminus of ERα (Lannigan 2003). Therefore, because this region is not required for activity, phosphorylation of the A/B domain of ER may not play a role in estrogenic activity of E_2_ or diarylheptanoids in HepG2 cells. The role of AF2 in ER activity is also cell-type specific (Tzukerman et al. 1994). In contrast, partial deletion of various ER regions has indicated that the AF2 region of ER is required for hormone-dependent transcription (Danielian et al. 1992). Here, we constructed an ER with point mutations at L543A/L544A that resides in the AF2 region on helix 12; because helix 12 of the ligand-binding domain forms part of a hydrophobic cavity of the receptor that is crucial for E_2_ agonist binding (Brzozowski et al. 1997), this mutation dampened the transcriptional activity of E_2_ as well as the diarylheptanoids. Therefore, these hydrophobic amino acids (L543 and L544) would appear to be involved in the inter action between diarylheptanoids and the ER, as is also seen with other ER agonists. We therefore surmise that diarylheptanoids behave more like agonists than antagonists, as shown by these responses that are similar to those of E_2_.

The classical uterine bioassay, which measures any increase in uterine weight that results from administration of a test substance, is a useful tool for determining estrogenicity (Owens and Ashby 2002). In the present study, D3 caused a marked increase in uterine weight, although the increase was less than that of E_2_. Interestingly, the uterotrophic response to D3 proved to be ER dependent because it was essentially inhibited by ICI, a specific ER antagonist, and we observed no activity in αERKO mice. In addition, AR is also expressed in uterine tissues, and androgen-specific ligands, such as 5α-dihydrotestosterone or synthetic androgen, can increase uterine weight. Flutamide, an AR antagonist, inhibits the response (Hewitt et al. 2006). However, the uterine action of diarylheptanoid was not mediated through AR because flutamide did not inhibit the increased uterine weight. These findings confirmed that the estrogenic activity of diaryl heptanoid was not through the AR.

The stimulatory effect of D3 on the proliferation of uterine epithelium was further supported by anti-Ki67 immunohistochemistry staining, which detects a nuclear antigen present only in proliferating cells (S, G_2_, and M phase) (Gerdes et al. 1984). We found positive staining of anti-Ki67 within the uterine epithelium in mice treated with E_2_, D3, or D3 together with flutamide. Therefore, D3 exhibited a proliferative effect similar to E_2_ on mouse uterine epithelial cells.

Several studies have shown that Ltf, an iron-binding glyco protein (Teng 2002), is an E_2_-inducible gene in uterine epithelium in mice (Hewitt et al. 2003), rats (Jefferson et al. 2000), monkeys, and humans (Teng et al. 2002). Ltf is also present in most exocrine secretions and in the secondary granules of polymorphonuclear leucocytes (Walmer et al. 1992). The expression of mouse Ltf in the genital tract is correlated with rising serum estradiol concentration because the protein has been detected at early and late estrous during natural estrous cycle (Walmer et al. 1992). Moreover, an ERE is located on the promoter of the *Ltf* gene (Ray et al. 2006). In the present study, the D3–up-regulated *Ltf* expression was similar to that of estrogen but less robust. In addition, the *Ltf* expression pattern correlated well with the increase in uterine weight. Use of selective antagonists showed the effect of D3 on *Ltf* expression was also ER dependent, not mediated through AR. Although D1 and D2 showed estrogenic activity in *in vitro* experiments that was similar to D3, neither D1 nor D2 was effective in stimulating uterine response. This may be due to metabolic inactivation of D1 and D2 in the whole mouse. Therefore, estrogenicity of compounds should be evaluated in both *in vivo* and *in vitro* models to allow the full assessment and appreciation of the exact biological responses that could be expected with human exposures.

We then evaluated in more detail the different potential mechanisms of action of D3 through both classical and nonclassical ER pathways. In the classical pathway, E_2_–ER binds directly to ERE DNA motifs in genes (ERE dependent), whereas ERα can interact with other DNA-binding transcription factors by protein–protein interaction (ERE independent) (Hall et al. 2001). The KI/KO mice were previously used to elucidate the *in vivo* ERE-independent signaling of estrogen because they lacked any ERE binding activity due to a DNA binding domain mutation of ERα (O’Brien et al. 2006). Increase in *Aqp5* transcript required the ERE-mediated pathway, whereas increase in *Cdkn1a* (p21) could be regulated by the ERE-independent mechanism (O’Brien et al. 2006). Moreover, the ERE element in the 5′ region of *Aqp5* was directly regulated by E_2_–ERα (Kobayashi et al. 2006). Herein, we demonstrated that *Aqp5*, one of the important genes for fluid absorption, was up-regulated by D3 and E_2_ only in WT mice. We also demonstrated D3 was able to up-regulate *Cdkn1a* both in WT and KI/KO, which implied that D3 is able to exert estrogenic activity through either classical or ERE-independent mechanisms *in vivo. Aqp5* and *Cdkn1a* were not up-regulated by D3 and E_2_ in αERKO mice, indicating the ER-dependence of these genes’ activation.

In conclusion, the present study suggests that diaryl heptanoids (D1, D2, and D3) exhibit estrogenic properties selectively through ERα, particularly requiring AF2. Moreover, the estrogenic activity of D3 can involve direct DNA binding or ERE-independent gene regulation both *in vitro* and *in vivo*. This is the first report on the molecular mechanism underlying the action of diarylheptanoid, the major compound in the environmental phytoestrogen *C. comosa*, thereby confirming estrogenic activity, which provides evidence and explanation for therapeutic and exposure effects and potential further development. Because the *in vivo* actions of diarylheptanoid (D3) appear to be selective for ERα, the potential target tissues that can be affected include any tissues that predominantly express ERα, not merely the reproductive system. Evidence of primary physiologic roles and actions for ERα is exemplified from studies involving the ER knockout mouse models (Couse and Korach 1999) and a patient with an ERα inactivation mutation (Smith et al. 2008).

Another issue of concern is that plants such as *Curcuma longa* (turmeric), which contain diarylheptanoids, have been used extensively for cancer chemoprevention. Surprisingly, none of the diaryl heptanoid cancer prevention studies have evaluated effects in the reproductive system. Our study provides evidence that diarylheptanoids exhibit sex-steroid (estrogen)-like activity *in vivo*. Additionally, we report *in vitro* evaluation of possible mechanisms of action. Our findings may evoke public interest concerning any hormonal disturbance resulting from use or exposure to such plant-derived products and add further evidence to the investigation of the sources and chemical nature of phytoestrogens.

## Figures and Tables

**Figure 1:**
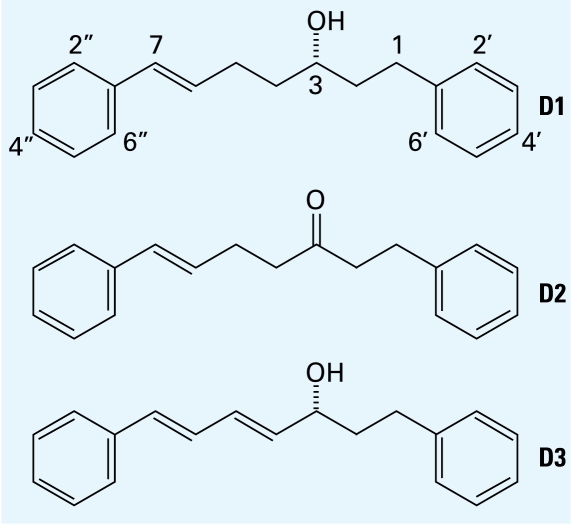
Structure of the diarylheptanoids (3*S*)-1,7-diphenyl-(6*E*)-6-hepten-3-ol (D1), 1,7-diphenyl-(6*E*)-6-hepten-3-one (D2), and (3*R*- ) 1,7-diphenyl-(4*E*,6*E*)-4,6-heptadien-3-ol (D3), purified compounds isolated from rhizome (Suksamrarn et al. 2008).

**Table 1:** Human and mouse primers.

Gene symbol (complete name; GenBank accession number)	Sequence (5′→ 3′)
*Aqp5* (aquaporin 5; NM_009701.4)	F: ACAGGGCCTCTTTGGAATTAGG
	R: TGAGCTTGCACTGCCTTCAC
*B2M* (beta-2-microglobulin; NM_004048.2)	F: TGCTCGCGCTACTCTCTCTTT
	R: TCTGCTGGATGACGTGAGTAAAC
*Cdkn1a* (cyclin-dependent kinase inhibitor 1A; NM_007669.4)	F: CAGCGACCATGTCCAATCC
	R: CGAAGAGACAACGGCACACTT
*CTSD* (cathepsin D; NM_001909.3)	F: ACTGCTGGACATCGCTTGCT
	R: TGTCAAACGAGGTACCATTCTTCA
*Ltf* (lactotransferrin; NM_008522.3)	F: CAGCAGGATGTGATAGCCACAA
	R: CACTGATCACACTTGCGCTTCT
*MYC* (v-myc myelocytomatosis viral oncogene homolog; NM_002467.3)	F: TGCCGCATCCACGAAACT
	R: GTCCTTGCTCGGGTGTTGTAAG
*PGR* (progesterone receptor; NM_000926.4)	F: GACGTGGAGGGCGCATAT
	R: AGCAGTCCGCTGTCCTTTTCT
*Rpl7* (ribosomal protein L7; NM_011291.4)	F: AGCTGGCCTTTGTCATCAGAA
	R: GACGAAGGAGCTGCAGAACCT
*TFF1* (trefoil factor 1;pS2 NM_003225.2)	F: GCCCTCCCAGTGTGCAAATA
	R: CTGGAGGGACGTCGATGGTA

Abbreviations: F, forward; R, reverse. The primer sequences were created using Applied Biosystems Primer Express Software version 2.0. All gene names and accession number were obtained from GenBank (National Center for Biotechnology Information 2009).
